# What Keeps Older People out of Nursing Homes? A Meta-Analysis

**DOI:** 10.1093/geroni/igab046.1963

**Published:** 2021-12-17

**Authors:** Joseph Gaugler, Rachel Zmora, Colleen Peterson, Lauren Mitchell, Robyn Birkeland, Eric Jutkowitz, Sue Duval

**Affiliations:** 1 University of Minnesota, Minneapolis, Minnesota, United States; 2 University of Minnesota, University of Minnesota, Minnesota, United States; 3 Emmanuel College, Boston, Massachusetts, United States; 4 Brown University, Brown University, Rhode Island, United States

## Abstract

Perhaps one of the most examined, and costly, health transitions older people experience is nursing home admission. In addition to the financial costs nursing home admission poses to older people, their families, and other payers (e.g., the public), institutionalization is linked with a range of negative outcomes and represents a loss of independence and quality of life to many older persons. The current meta-analysis attempted to synthesize all available randomized controlled trials available to ascertain which intervention approaches appeared to prevent nursing home entry for older adults. The MEDLINE, PsycInfo, CINAHL, Cochrane, and EMBASE databases were searched to August, 2020. Abstracts were screened (N = 28,120) to identify randomized controlled trials of interventions to prevent or delay nursing home admission as well as systematic reviews. Identified studies were cross-referenced until the point of saturation, resulting in 1,786 studies for additional inclusion/exclusion screening. Following a consensus-based review among the authors that included risk of bias, 323 randomized controlled trials were included in the meta analysis. Although several intervention modalities appeared protective against nursing home admission and approached statistical significance, preliminary results suggest that comprehensive geriatrics assessment (pooled OR = .69, 95% CI: .50, .95) and specialized, inpatient geriatrics care (pooled OR: .77, 95% CI: .59, .99) were most consistent in helping to prevent institutionalization among older persons. The findings emphasize the importance of geriatrics when delivering optimal care to older persons. Integrating such approaches more effectively into a largely fee-for-service healthcare paradigm remain a critical challenge.

